# A novel digital health approach to improving global pediatric sepsis care in Bangladesh using wearable technology and machine learning

**DOI:** 10.1371/journal.pdig.0000634

**Published:** 2024-10-30

**Authors:** Stephanie C. Garbern, Gazi Md. Salahuddin Mamun, Shamsun Nahar Shaima, Nicole Hakim, Stephan Wegerich, Srilakshmi Alla, Monira Sarmin, Farzana Afroze, Jadranka Sekaric, Alicia Genisca, Nidhi Kadakia, Kikuyo Shaw, Abu Sayem Mirza Md. Hasibur Rahman, Monique Gainey, Tahmeed Ahmed, Mohammod Jobayer Chisti, Adam C. Levine

**Affiliations:** 1 Department of Emergency Medicine, Warren Alpert Medical School, Brown University, Providence, Rhode Island, United States of America; 2 International Centre for Diarrhoeal Disease Research, Bangladesh (icddr,b), Dhaka, Bangladesh; 3 PhysIQ, Inc. Chicago, Illinois, United States of America; 4 Brown University, Providence, Rhode Island, United States of America; Arak University of Technology, ISLAMIC REPUBLIC OF IRAN

## Abstract

Sepsis is the leading cause of child death globally with low- and middle-income countries (LMICs) bearing a disproportionate burden of pediatric sepsis deaths. Limited diagnostic and critical care capacity and health worker shortages contribute to delayed recognition of advanced sepsis (severe sepsis, septic shock, and/or multiple organ dysfunction) in LMICs. The aims of this study were to 1) assess the feasibility of a wearable device for physiologic monitoring of septic children in a LMIC setting and 2) develop machine learning models that utilize readily available wearable and clinical data to predict advanced sepsis in children. This was a prospective observational study of children with sepsis admitted to an intensive care unit in Dhaka, Bangladesh. A wireless, wearable device linked to a smartphone was used to collect continuous recordings of physiologic data for the duration of each patient’s admission. The correlation between wearable device-collected vital signs (heart rate [HR], respiratory rate [RR], temperature [T]) and manually collected vital signs was assessed using Pearson’s correlation coefficients and agreement was assessed using Bland-Altman plots. Clinical and laboratory data were used to calculate twice daily pediatric Sequential Organ Failure Assessment (pSOFA) scores. Ridge regression was used to develop three candidate models for advanced sepsis (pSOFA > 8) using combinations of clinical and wearable device data. In addition, the lead time between the models’ detection of advanced sepsis and physicians’ documentation was compared. 100 children were enrolled of whom 41% were female with a mean age of 15.4 (SD 29.6) months. In-hospital mortality rate was 24%. Patients were monitored for an average of 2.2 days, with > 99% data capture from the wearable device during this period. Pearson’s r was 0.93 and 0.94 for HR and RR, respectively) with r = 0.72 for core T). Mean difference (limits of agreement) was 0.04 (-14.26, 14.34) for HR, 0.29 (-5.91, 6.48) for RR, and -0.0004 (-1.48, 1.47) for core T. Model B, which included two manually measured variables (mean arterial pressure and SpO2:FiO2) and wearable device data had excellent discrimination, with an area under the Receiver-Operating Curve (AUC) of 0.86. Model C, which consisted of only wearable device features, also performed well, with an AUC of 0.78. Model B was able to predict the development of advanced sepsis more than 2.5 hours earlier compared to clinical documentation. A wireless, wearable device was feasible for continuous, remote physiologic monitoring among children with sepsis in a LMIC setting. Additionally, machine-learning models using wearable device data could discriminate cases of advanced sepsis without any laboratory tests and minimal or no clinician inputs. Future research will develop this technology into a smartphone-based system which can serve as both a low-cost telemetry monitor and an early warning clinical alert system, providing the potential for high-quality critical care capacity for pediatric sepsis in resource-limited settings.

## Author summary

Sepsis, a condition that arises when the body’s response to an infection injures its own tissues and organs, is the leading cause of child death globally. Limited diagnostic and critical care capacity and health worker shortages in low resource settings contribute to delayed recognition of sepsis and an increase in deaths among children. The aims of this study were to 1) assess the feasibility of a wearable device for monitoring sepsis in children in a low resource setting and 2) develop machine learning models that utilize readily available wearable and clinical data to predict sepsis in children. This was a prospective study of children admitted to an intensive care unit in Dhaka, Bangladesh. A wireless, wearable device linked to a smartphone was used to collect continuous recordings of physiologic data for the duration of each patient’s admission. 100 children were enrolled and monitored for an average of 2.2 days, with > 99% data capture from the wearable device. There was high agreement between the vital signs measured by the wearable device and those measured by clinicians. Models developed using data collected from the device were able to predict sepsis in children with moderate accuracy hours earlier than clinicians caring for the patients. Future research will develop this technology into a smartphone-based system, which can serve as a low-cost telemetry monitor and an early warning system in low resource settings.

## Introduction

Sepsis, defined as life-threatening organ dysfunction caused by a dysregulated host response to infection, is the leading cause of child mortality worldwide. Low- and middle-income countries (LMICs) bear a disproportionately high burden of sepsis, stemming from infections such as pneumonia, diarrhea, and malaria [1–5]. Mortality remains substantially greater for children in LMICs compared to high-income countries (HICs) for a multitude of reasons including: inadequate critical care capacity, shortages of trained healthcare workers (HCWs), higher levels of co-infections and malnutrition, as well as late identification of sepsis [4,6].

Sepsis encompasses a continuum of severity that ranges from sepsis to severe sepsis, septic shock, multiple organ dysfunction syndrome (MODS) and eventually death if untreated [3,7,8]. Management of septic children is challenging even for experienced clinicians, as children often have dynamic and subtle physiologic changes which indicate worsening sepsis [4,8]. These challenges are compounded in settings with limited human and infrastructural resources for monitoring and diagnosis [4,8]. Frequent, accurate assessments of sepsis severity are important for effective patient management, allowing clinicians to intervene earlier, select the most appropriate treatments, and optimally utilize scarce healthcare resources to reduce morbidity and mortality [9].

However, there remains a paucity of research from LMICs regarding the best methods for assessing sepsis severity for risk stratification and better directing care. While clinical scoring systems such as the pediatric logistic organ dysfunction (PELOD-2), pediatric sequential organ dysfunction assessment (pSOFA) scores, and the recent Phoenix Sepsis Score have been used largely in HICs, these scores are often not feasible to operationalize in LMICs due to their reliance on regular laboratory tests, clinical assessments from experienced HCWs, and relatively cumbersome calculations [4,10–12]. Furthermore, while continuous telemetry monitoring is standard practice in HICs for sepsis care, in many LMICs, even intermittent vital sign checks to detect signs of progressive sepsis can place a substantial burden on HCWs with high patient-to-clinician ratios.

**Fig 1:**
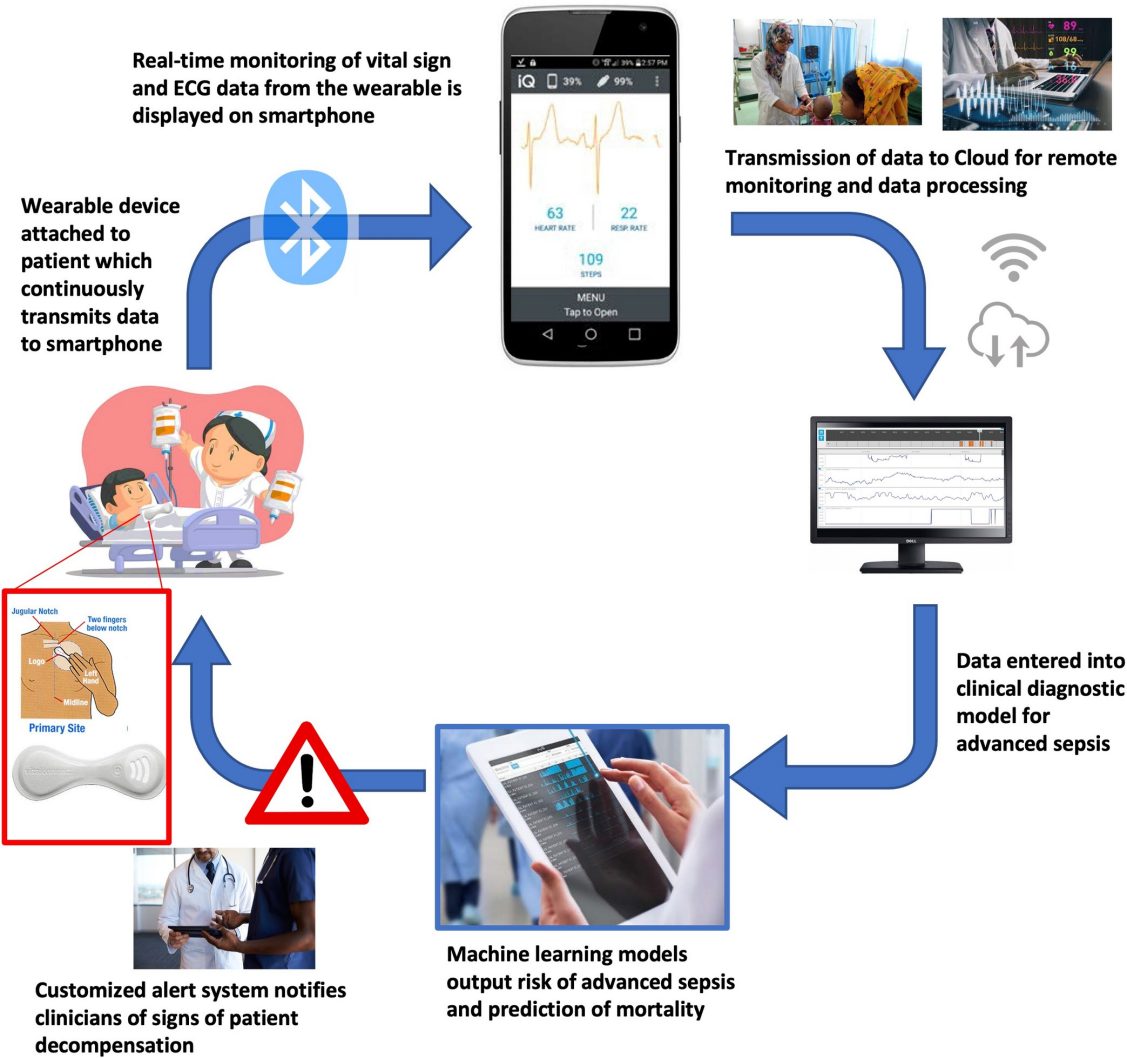
Schematic of wearable-enabled mobile health technology for improved pediatric sepsis care.

Innovative digital technologies hold great potential to overcome some of the greatest barriers to improving sepsis care. Mobile health (mHealth) clinical decision support systems (CDSS), wearable devices, and artificial intelligence (AI) techniques have rapidly proliferated for a multitude of inpatient clinical applications, although few studies have been done in LMICs to date [13]. The objectives of this study were to 1) determine the feasibility of using a wireless, wearable device for continuous remote vital sign monitoring in children with sepsis and 2) develop a clinical diagnostic model using primarily wearable device data to predict advanced sepsis (infection with organ dysfunction) among children in Bangladesh. The findings from this research will be used to develop this technology into a mHealth system which serves as a low-cost, remote telemetry monitor and CDSS, improving critical care capacity for pediatric sepsis in resource-limited settings (Fig 1).

## Materials and methods

### Study design and setting

This was a prospective, observational study of children with suspected sepsis who were admitted to the International Centre for Diarrhoeal Disease Research, Bangladesh (icddr,b) Dhaka Hospital Intensive Care Unit (ICU). Dhaka Hospital provides free clinical services to >200,000 patients annually from a catchment area of more than 17 million people. Data were collected from February–December 2022. Due to an unprecedented outbreak of cholera, study activities were briefly paused in April 2022 as staff were diverted to assist with clinical care of patients. Ethical approval was obtained from the icddr,b Institutional Review Board comprising Research Review Committee and Ethical Review Committee and the Rhode Island Hospital Institutional Review Board.

### Study participants

All patients between two months (prematurity-corrected) to 17 years of age admitted to the ICU with suspected sepsis were eligible for enrollment. Suspected sepsis was defined as meeting at least two systemic inflammatory response syndrome (SIRS) criteria with suspected source of infection as determined by the treating physician, based on clinical practice during the enrollment period. Briefly, SIRS was defined according to Goldstein criteria as the presence of at least two of the following four criteria (one of which must be abnormal temperature or leukocyte count): 1) core temperature >38.5C or <36C; 2) tachycardia defined as mean heart rate (HR) >2 SD above normal for age or bradycardia <10^th^ percentile for age in children < 1 year; 3) mean respiratory rate (RR)>2 SD above normal for age or use of mechanical ventilation; 4) leukocyte count elevated or depressed for age [7]. Trained study staff screened all pediatric patients consecutively for enrollment upon ICU admission 24 hours/day, 7 days/week. To ensure high-quality data collection and adherence to study protocols, a maximum of three simultaneous patients were enrolled. After screening, the parent/guardian of eligible patients was offered participation and informed consent (and assent for patients 11–17 years) was obtained in the local language, Bangla.

### Staff training

A two-week pre-implementation phase and staff training period was conducted to ensure seamless integration of the wearable device monitoring system into clinical workflow prior to patient enrollment using a combination of didactics and hands-on training. Training focused on: 1) hands-on practice with the technology among all research staff (application, removal, replacement, and troubleshooting of the wearable device, smartphone operation, data quality monitoring, etc.) 2) logistics of implementing the technology including ensuring electrical and internet connectivity 3) collection of all clinical and laboratory data and assessments of sepsis severity. Dedicated research nurses and physicians were hired and trained to screen and enroll patients, obtain informed consent, collect data, and monitor data quality.

### Study procedures and data collection

**Fig 2:**
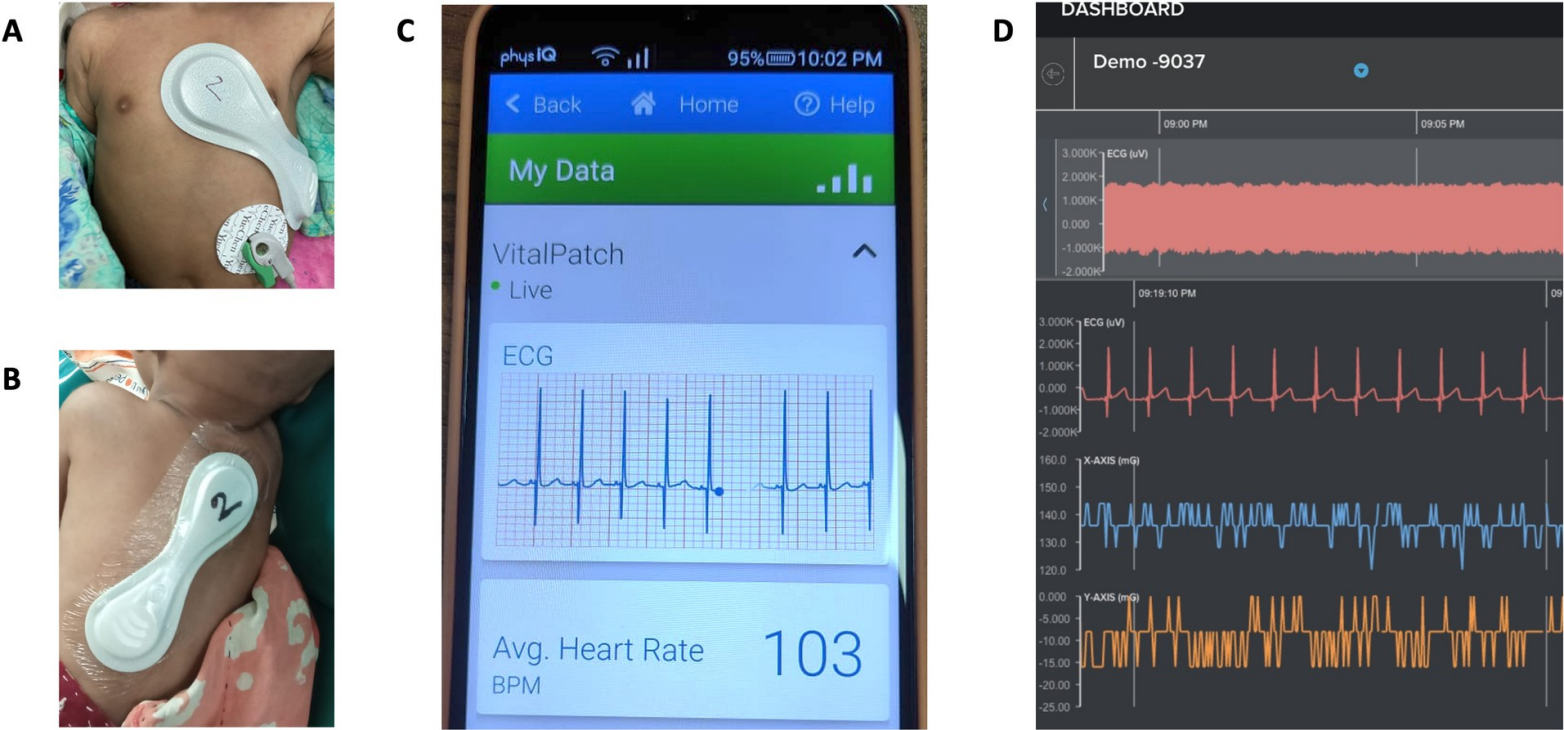
Overview of wearable monitoring technology. The VitalPatch wearable device was attached using a) standard anterior torso position for most patients b) posterior torso position for very small infants with transmission via Bluetooth to c) smartphone and d) browser-based remote monitoring dashboard.

After patient enrollment, study staff collected demographic and clinical data on all patients, including age, sex, height, weight, and mid-upper arm circumference (MUAC). Staff then applied a VitalPatch (VitalConnect, Inc., San Jose, CA), a medical grade wearable biosensor device, to the patient’s torso. The lightweight (13g), water-resistant, flexible device was applied via the device’s adhesive after cleaning the skin with an alcohol pad. An anterior chest wall position was used for most patients; for very small children (roughly <4kg) a posterior chest wall position was used as it was found this improved adhesion to the chest (Fig 2A and 2B). The device continuously transmitted the following data: single-lead ECG signal, three-dimensional accelerometer, uncalibrated skin temperature via Bluetooth to a connected Android (Samsung Galaxy A10e) smartphone (Fig 2C) which then transmitted data continuously to the remote Cloud servers run by PhysIQ, Inc. Real-time ECG and vital sign data were accessible to remote study investigators via a web browser-based monitoring dashboard (Fig 2D). Raw biosignal waveform data from the device was extracted and analyzed to generate the following parameters at one-minute intervals for the duration of the patient’s enrollment: HR, RR, heart rate variability (HRV), and skin and core temperature (T). Devices were removed at patient discharge by slowly peeling off the device from the skin.

Laboratory testing including complete blood count, electrolytes, glucose, renal function, hepatic function panel, lactate, blood gas, and coagulation panel were obtained upon admission, then twice daily (every 12 hours approximately). For patients with prolonged ICU admissions (four or more days), laboratory testing was reduced to once daily as deemed appropriate and according to clinical necessity by the treating physicians. Clinical exams and vital sign assessments were documented by study staff every hour including Glasgow Coma Score (GCS), manual vital signs (BP, HR, RR, T, SpO2), capillary refill time, and urine output, for determination of sepsis state using the Goldstein criteria and pSOFA score [7,11]. Patients enrolled in the study received routine care for sepsis according to institutional protocols which included clinical examinations, radiographs and ultrasound, administration of IV fluids, antibiotics, vasopressors, blood products, and use of bubble continuous positive airway pressure (CPAP) and mechanical ventilation as per standard guidelines followed in the ICU [14–16]. All data obtained during routine clinical care were entered on case report forms (CRF) separate from usual icddr,b clinical documentation.

### Outcomes

The primary outcome of interest was the development of advanced sepsis defined as pSOFA score > 8 (indicative of organ dysfunction and/or septic shock) calculated twice daily (approximately every 12 hours) by a research physician after the conclusion of the study. Researchers were thus blinded to the primary outcome during the conduct of the study. This outcome was chosen given its indication of clinically actionable sepsis states which often warrant higher level of care or more intensive treatments (e.g., vasopressors in shock). Additionally, calculation of pSOFA scores twice daily increased the number of outcomes available given the relatively small cohort. As a secondary outcome, we compared the time to recognition of advanced sepsis as determined using the clinical model compared to standard clinical documentation. In addition, we evaluated the feasibility, quality, correlation, and agreement of data collected from the wearable device, as described below.

### Data quality and capture

“High quality” data was assessed using an ECG signal quality index (SQI), which assesses signal strength, motion artifact content and noise levels over one-minute windows resulting in a quality metric ranging from 0–100% for each window. If the metric fell below 75%, data from the corresponding one-minute window was deemed “low quality” and not able to be used for subsequent data analysis. Data quality was assessed daily whenever there were active patients enrolled in the study.

### Feasibility

To assess feasibility, the following data was collected for each patient via the biosensor system or documentation on the CRF by study staff during regular checks: % of successfully transmitted data for each vital sign parameter; % of patients transmitting data within 30 minutes of enrollment; % of patients monitored for the entirety of their ICU stay; incidence and reasons for Bluetooth/internet connectivity problems, biosensor detachment/replacement, and/or battery charging problems.

### Comparison between manual and wearable-collected vital signs

To assess the ability of the system to support real-time, remote physiologic monitoring in a real-world clinical setting, vital signs collected using the smartphone-linked wearable device were compared to manual vital signs obtained by a research nurse (considered the gold standard in this setting). Comparisons were made between vital signs collected within the same five-minute period to accommodate variations in when the study nurse recorded the vital sign value and when they measured it. HR and RR were counted manually over 60 seconds by an experienced research nurse. Temperature was measured rectally for children under five years and orally for those over five years. Comparison between the wearable vital signs and manual vital signs was assessed using Pearson’s correlation coefficients and Bland-Altman plots accounting for repeated measures with mean differences +/- 1.96SD (also known as “limits of agreement”)[17].

### Data analysis and model development

Variables were described using frequencies with percentages, means with standard deviations (SD), or medians with associated interquartile ranges (IQR) as appropriate. Only clinical variables (i.e., those that would be readily available in resource-limited settings) and wearable device-collected variables were considered as candidate features for entry into the models as listed in Table 1. Laboratory values or clinical data not readily measured in resource-limited settings were not considered for model development. The Modified Pediatric Early Warning Score (MPEWS), a simplified early warning score used to monitor clinical deterioration in hospitalized children, was used to determine the most abnormal HR, RR, T, and SpO2 thresholds (“red zone”, the highest score according to MPEWS) (S1 Table) according to age as well as level of consciousness (AVPU [alert, voice, pain, unresponsive] criteria)[18].

**Table 1:** Candidate features for entry into prediction models.

**Manual Features**	Age (months), sex, presence of diarrhea, capillary refill*, peripheral edema, jaundice, delayed skin pinch, sunken eyes, pupillary reaction, general appearance, malnutrition (using mid-upper arm circumference [MUAC] or body mass index), weight (kg), height (cm), mean arterial pressure, SpO2:FiO2
**Wearable Device (VitalPatch)–Derived Features**	Mean and standard deviation (SD): worst (most extreme) respiratory rate, heart rate, heart rate variability, skin temperature, core temperature, and activity† Percent of time in the “danger” (red) zone for heart rate, respiratory rate, skin temperature, and core temperature using age-specific thresholds based on the modified pediatric early warning score (MPEWS) thresholds [18].

* A measure of peripheral perfusion

† Activity is a measure of movement as sensed by the VitalPatch triaxial accelerometer. It is the standard deviation of the 3D acceleration sensor data magnitude in 30-second windows, calculated every 5-seconds. The 5-second results are then averaged to produce minute values of “Activity”.

Note: Mean, SD, and worst vital sign variables were calculated from a one-hour period surrounding the time that study staff collected the laboratory tests and performed the clinical assessments used to calculate the pSOFA score.

Three candidate models were developed using Ridge regression (L2 regularization, a method that uses a penalty term to shrink coefficient values of less important features and reduce overfitting) to detect advanced sepsis, as defined using a pSOFA score > 8. The pSOFA score threshold of 8 has previously been validated to discriminate risk of mortality in the original validation study of the pSOFA score, and correlates with the presence of shock and/or MODS [11]. Model A was exploratory to identify features most correlated with the outcome, while the focus of Model B and C was to create a model with primarily wearable or wearable-only features, as these would be the most clinically useful in resource-limited settings (given they require only a few or no clinical inputs). Laboratory tests were not considered for inclusion in the models given the desire to create a model which would be usable in resource-limited settings where laboratory tests and critical care support are not uniformly available. The three candidate models included the following features which were selected *a priori* (see Table 1 for list of features):

- **Model A.** All features from Table 1 (i.e., wearable device and manually collected features)
- **Model B.** Best correlated features (all features which explained at least 4% of the outcome variation from Model A)
- **Model C.** Best correlated features collected from the wearable device (no manually collected data)

Model discrimination was assessed using area under the receiver-operator characteristic curve (AUC). Each model was trained and tested on data from the first 12 hours after study enrollment, using 5-fold cross validation iterated 1,000 times. R and STATA Version 17 (Stata Corp; College Station, USA) were used for analysis. Transparent Reporting of a Multivariable Prediction Model for Individual Prognosis or Diagnosis (TRIPOD) guidelines were followed for model development and reporting [19].

### Secondary analyses

As secondary outcomes, the ability of the best performing model (Model B) to detect advanced sepsis (septic shock and/or MODS) was compared to physician diagnoses, using clinical documentation from the icddr,b Dhaka Hospital SHEBA electronic health record (EHR) completed as part of standard-of-care. Clinicians’ (non-study physicians) assessments (as documented in the EHR) were categorized as sepsis (i.e., without features of MODS or shock) or advanced sepsis (i.e., with septic shock and/or MODS) for comparison against Model B. The false positive rate for clinician estimates of the presence of advanced sepsis was compared to our criterion standard of a pSOFA score > 8 in order to determine the threshold at which to assess the sensitivity and F1 score (machine learning metric that combines sensitivity and positive predictive value for model classification performance) of our model and compare those to the sensitivity and F1 score of the clinicians. Using a one-sample two-sided t-test, the model and clinicians’ sensitivity, accuracy, and F1 scores were calculated across 1,000 model iterations.

Additionally, the time when Model B could detect advanced sepsis was compared to clinical documentation. To assess this, two analyses were conducted: 1) the “lead times” between detection of advanced sepsis using Model B or clinical documentation against the twice daily pSOFA calculations that were obtained from all study patients (at approximately 8am and 8pm each day) using Mann-Whitney U test. Lead times were calculated as the difference in time between the first pSOFA score > 8 and the first diagnosis of advanced sepsis from Model B or the timestamped EHR clinical documentation 2) the lead times between when pSOFA was > 8 after previously being < 8 (an indication of progressive sepsis) as determined by Model B versus clinical documentation. To calculate and compare lead times, the model was iterated 1000 times, with data randomly segmented into training and testing sets. The lead times were averaged from each patient across all iterations resulting in one lead time per patient. Comparison of model versus clinician documentation lead times was then conducted using the Mann-Whitney U test.

### Sample size

In order to show a significant difference between an AUC of at least 0.7 (often used as the minimum discrimination for a model that is clinically meaningful) and the null (AUC of 0.5, no discrimination ability), at least 20 patients with severe sepsis (pSOFA > 8) were required using a power of 0.90 and alpha of 0.05 using standard calculations [20,21]. Based on prior research at the study site, approximately 20–25% of pediatric sepsis patients admitted to the ICU had septic shock, therefore a target sample size of 100 patients was used.

## Results

### Enrollment and population characteristics

**Fig 3:**
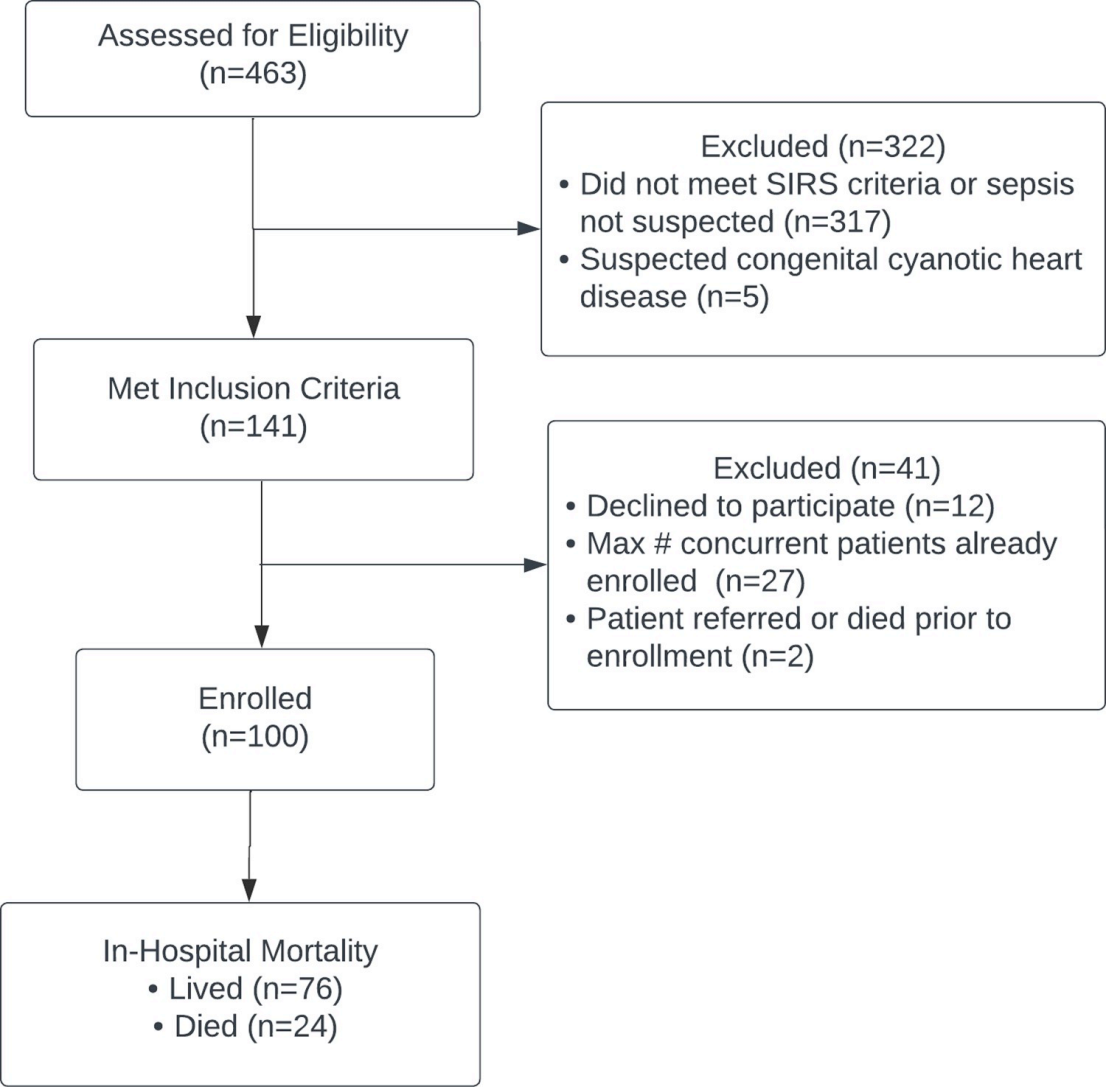
Study flow diagram.

The study flow diagram is shown in Fig 3. A total of 100 children were enrolled of whom 41% were female with mean age of 15.4 (SD 29.6) months. All enrolled patients completed the study protocol; there were no study withdrawals. In-hospital mortality was 24%, in line with prior mortality rates from the same study site [22]. Nearly half (46%) of patients were also diagnosed with severe acute malnutrition during their hospitalization. Table 2 shows further descriptive characteristics of the study cohort.

**Table 2:** Descriptive characteristics of the study population.

Characteristic	n (%) (N = 100)
Age (months)*	8 [5–18]
Sex	
Female	41 (41)
Male	59 (59)
Duration of Current Illness (days)*	4 [2–6.25]
Referred from another medical facility	
Yes	21 (21)
No	79 (79)
Past Medical History^	
None Known	84 (84)
Developmental Disability	5 (5)
Cerebral Palsy	5 (5)
Heart Disease (non-cyanotic)	2 (2)
Premature Birth	2 (2)
Epilepsy	1 (1)
Cleft Palate	1 (1)
Monthly Household Income (Taka / $USD)	Tk 15,000 [10,000–20,000] / $137 [91–182]
Years of Mothers’ Education^†^*	6.5 [5–9.25]
Preliminary Source(s) of Sepsis^^^	
Gastrointestinal / Diarrheal	98 (98)
Respiratory / Pneumonia	54 (54)
Central Nervous System / Meningitis	15 (15)
Genitourinary	2 (2)
Bloodstream	2 (2)
Other / Unknown	1 (1)
Severe Acute Malnutrition	
Yes	46 (46)
No	54 (54)
Admission pSOFA Score	5 [3–7]
Hospital Length-of-Stay (days)*	6 [4–9.75]
In-Hospital Mortality	
Lived	76 (76)
Died	24 (24)

*Median [IQR = interquartile range]

†Mother or primary female caregiver

^ Multiple selections allowed therefore numbers do not sum to 100%

The median admission pSOFA score was 5 [3–7] and median admission PELOD-2 score was 3 [2–5]. Using the data collected twice-daily for each child during their ICU stay, we were able to calculate 417 pSOFA scores, out of which the rate of the primary outcome (pSOFA > 8) was 21% (88/417). pSOFA scores were missing for 9% of these twice-daily measurements (due to missing laboratory test results). There were no missing clinical predictor variables or biosensor data for the twice daily assessments.

### Data quality and capture

Patients were monitored for an average of 52.5 hours (2.2 days) with the wearable device, with a median of > 99% “high quality” data capture over each patient’s study enrollment period. Usable data excluded poor quality data due to artifact (e.g., due to patient movement), failure of transmission from device to Cloud server (e.g., poor internet connectivity), or periods of device disconnection between the device and the smartphone (e.g., for obtaining radiologic studies, non-adherence to skin, etc). There were no weeks during which data capture dropped below 98% over the study duration.

### Comparison between manual and wearable device-collected vital signs

**Fig 4:**
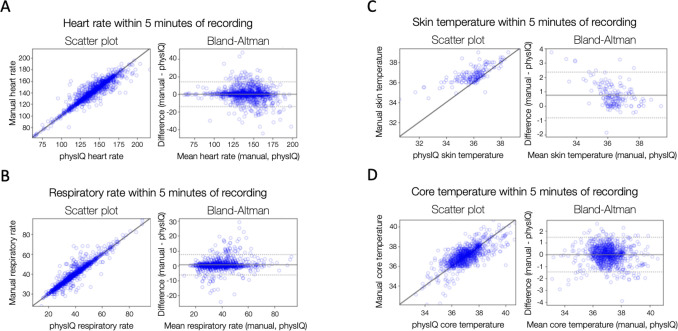
Correlation and Bland-Altman plots assessing correlation (left) and agreement (right) between vital signs collected manually versus via smartphone-linked wearable device (PhysIQ) for A) heart rate B) respiratory rate C) skin temperature and D) core temperature.

Wearable device-collected vital signs were compared to manual vital signs collected by experienced study nurses using Pearson’s correlation and Bland-Altman plots (Table 3 and Fig 4). Pearson’s correlation was excellent for heart rate (HR) and respiratory rate (RR) with Pearson’s r = 0.93 for HR and r = 0.94 for RR, as shown in Table 3. Correlation was less robust but still acceptable for core T (r = 0.72) and skin T (r = 0.74). Mean difference (limits of agreement) was 0.04 (-14.26, 14.34) for HR, 0.29 (-5.91, 6.48) for RR, -0.0004 (-1.48, 1.47) for core T, and 0.76 (-5.91, 6.48) for skin T.

**Table 3:** Comparison between manual and wearable-collected vitals using Pearson’s correlation and Bland-Altman statistics.

	Pearson’s r	Mean absolute error (MAE)	Bland-Altman Mean Difference [Bias] (+/- 1.96 SD [Limits of Agreement])
Heart rate (beats/minute)	0.93	4.17	0.04 (-14.26, 14.34)
Respiratory rate (respirations/minute)	0.94	1.53	0.29 (-5.91, 6.48)
Skin temperature (C°)	0.74	0.82	0.76 (-0.84, 2.36)
Core temperature (C°)	0.72	0.56	-0.0004 (-1.48, 1.47)

### Clinical diagnostic model for sepsis severity

**Fig 5:**
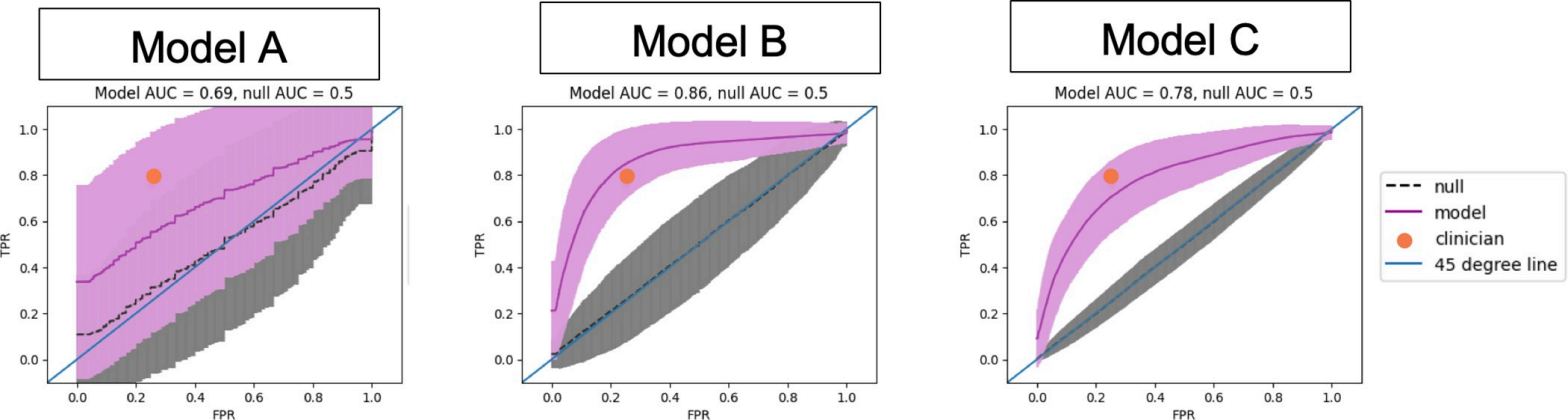
ROC curves from the three Ridge regression models developed with area under the ROC curve (AUC) indicated discrimination performance of the models. False positive rate (FPR); true positive rate (TPR). The dark purple line represents the mean ROC curve across all iterations, and the lighter-colored error bars represent ± 1 standard deviation. The orange dot represents the clinicians’ performance.

Of the three candidate models developed, Model B had the best discriminatory ability to detect pSOFA > 8 with AUC of 0.86, indicating excellent discriminatory ability, and a significant difference between the null and observed models (p < 0.02). Model B included the following features: mean arterial pressure (MAP), worst (highest or lowest) HR, SD of the RR, SpO2:FiO2, mean HR, worst RR. Model C, which consisted of only wearable device features (i.e., without any clinician-measured inputs), also performed well, with an AUC of 0.78 and a significant difference between the null and observed models (*p* < 0.009). Model C included the following features: mean HR, worst HR, SD of RR, worst RR, percent of time RR was in a severely abnormal range (using MPEWS thresholds), SD of skin T, mean core T. The discriminatory performance of each candidate model is shown in Fig 5. The mean AUC from Model A (all candidate features) was 0.69 with no significant difference between the null and observed models (*p* < 0.33).

### Secondary outcomes–comparison to clinician diagnosis and time to detection of advanced sepsis

The results of the comparison between Model B and clinician documentation of advanced sepsis are summarized in Table 4. Model B was more sensitive compared to clinicians in diagnosing advanced sepsis (mean model sensitivity = 0.83; clinician sensitivity = 0.76; p < 0.001), while equally specific (model and clinician specificity = 0.75).

**Table 4:** Comparison of clinicians versus Model B’s performance at diagnosing advanced sepsis states.

	Clinicians	Model B	Significance **(p<0.05)
False positive rate	0.25	0.25	t(988) = -0.90, p = 0.37
Sensitivity	0.76	0.83	t(988) = 15.55, p < 0.001**
Specificity	0.75	0.75	t(988) = 1.26, p = 0.21
Accuracy	0.75	0.76	t(988) = 4.64,p < 0.001**
F1 score	0.45	0.53	t(988) = 13.05, p < 0.001**

**Fig 6:**
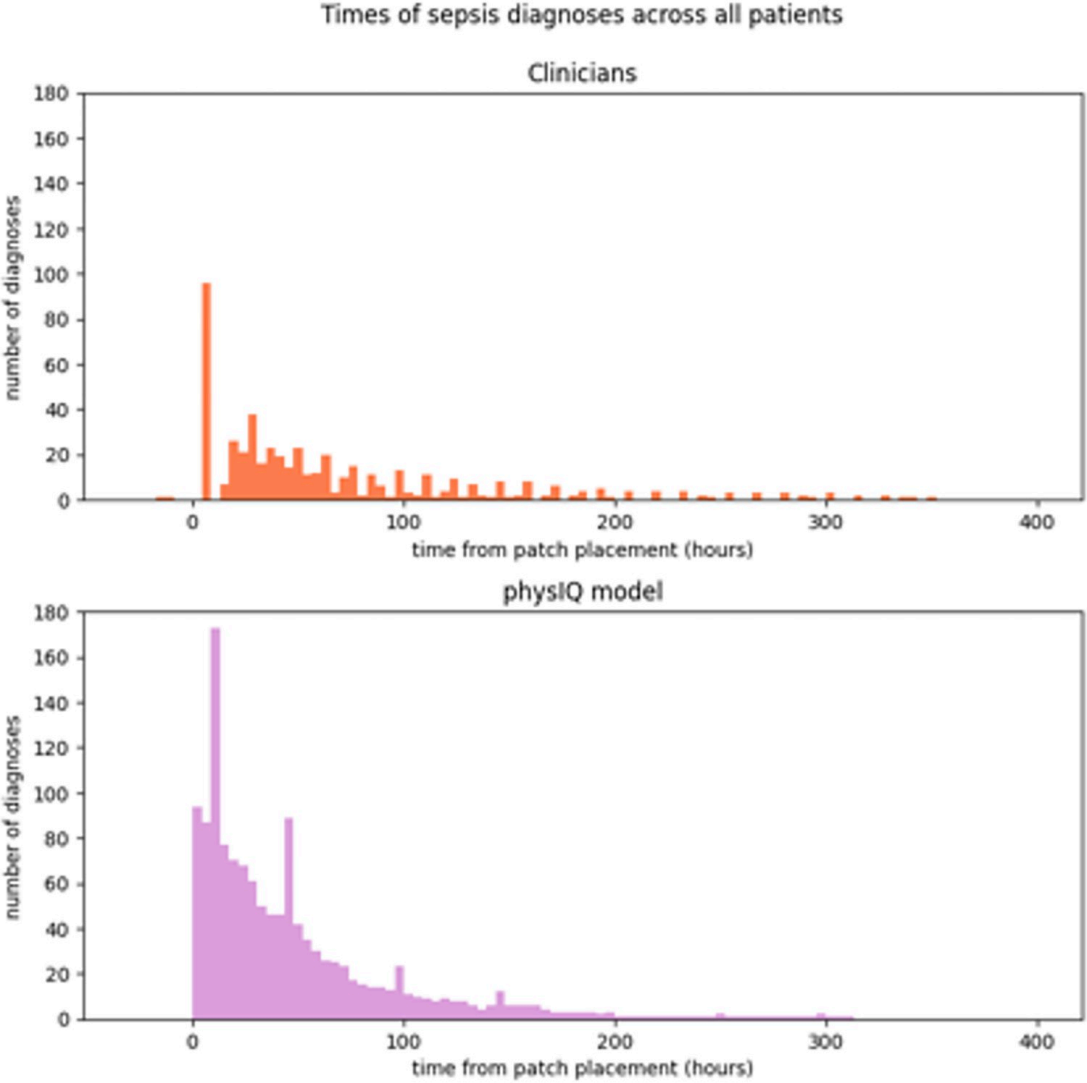
Distribution of times when the clinicians and Model B made first advanced sepsis diagnoses.

Fig 6 shows the distribution of times, across all patients, for Model B and clinician documentation of advanced sepsis diagnoses. The model made an average of 13.59 (SD 11.96) diagnoses per patient and the model’s first diagnosis was made on average 4.33 (SD 2.36) hours after the wearable device was placed on a patient. Clinicians made an average of 5.22 (SD 5.4) diagnoses per patient, with their first diagnosis made on average 6.26 (SD 2.70) hours after the wearable device was placed on a patient, approximately 2 hours later than the model. The model made more diagnoses compared to clinical documentation (model mean = 13.59; clinician mean = 5.22; p < 0.001). Additionally, Model B detected advanced sepsis significantly earlier than the clinicians’ diagnosis (model mean = 4.22 versus clinician mean = 6.26 hours after wearable device placement, p < 0.001).

The lead times between first diagnosis of advanced sepsis (pSOFA>8) and Model B and clinician documentation of advanced sepsis were 10.2 (SD 8.3) hours for Model B and 6.5 (12.9) hours for clinicians (p = 0.066), demonstrating Model B could detect sepsis earlier than what was documented in the EHR. In aggregated analysis, Model B’s lead times were significantly higher than the clinician lead times (model mean = 6.88 hours; clinician mean = 4.19 hours; p<0.001). This demonstrates that Model B was able to detect advanced sepsis earlier than that documented in the EHR clinical documentation.

### Feasibility of physiologic monitoring using wearables

After the training period, research staff were able to successfully integrate use of the wearable mobile device monitoring system seamlessly into routine clinical activities. Patients routinely received procedures such as radiographs, ultrasounds, intubation and mechanical ventilation, and standard telemetry monitoring without significant issues in data capture using the wearable monitoring system. The study team worked closely with the clinical team if removal/replacement of biosensor devices was needed for clinical procedures, with minimal disruptions in data collection.

Internet connectivity issues were infrequent (less than once per month on average during the course of patient enrollment) with use of 3/4G mobile network for nearly all data transmission. Additionally, during periods of poor connectivity, due to redundancy allowing for data storage on the wearable device itself for up to 18 hours, there was no data loss during brief periods of poor connectivity. Throughout the course of the study, connectivity issues were detected and resolved independently by icddr,b research staff within less than 1 hour on average. Additionally, US-based investigators were able to monitor patient enrollment and data collection remotely in real-time without issues.

## Discussion

This study has demonstrated that a novel, wearable-enabled, mobile monitoring system was feasible for continuous, remote physiologic monitoring among critically ill children with sepsis in an inpatient setting in urban Bangladesh. Additionally, the study shows that a clinical diagnostic model using data primarily collected from the wearable device could discriminate advanced sepsis cases without the use of any laboratory tests, and minimal or no clinician inputs making it particularly beneficial for use in resource-limited settings.

While further validation of these models and implementation research are still needed prior to use in real-world clinical settings in LMICs, these findings indicate the potential for wearables to help overcome some of the most challenging barriers to providing high-quality patient monitoring in resource-constrained clinical settings, where telemetry monitoring is not feasible for most patients. Furthermore, using machine learning (ML) methods, clinical diagnostic models for sepsis severity can be developed based entirely or mostly on wearable-derived features. These results show that this novel mHealth wearable monitoring system, combined with our clinical diagnostic model, may allow clinicians to rapidly discriminate cases of advanced sepsis among children with infectious diseases even without laboratory tests or prior to a trained physician assessing the patient.

The ability of our model to detect advanced sepsis at a similar, or earlier time, than the documentation of experienced ICU clinicians demonstrates the potential benefit that use of these models may have compared to the current standard care. Given the rapid deterioration that often precedes refractory shock and death in pediatric sepsis, even a few hours of lead time in alerting clinicians may allow clinicians to intervene with treatments such as antibiotics, intravenous fluids, mechanical ventilation, or vasopressors to avoid or minimize decompensation and death. Benefits may be even greater in lower-resourced health facilities, such as clinics or sub-district hospitals, where there are lower clinician-to-patient ratios and many fewer clinicians with specialized expertise in pediatric sepsis.

These models, if incorporated into a clinical decision support system (CDSS) run on a mobile device, could allow clinicians to view a patients’ risk of severe sepsis on demand, aiding them in making decisions and adhering to best practices regarding patient management. However, while CDSS have been widely used and shown to be effective in HICs, there remains a paucity of research on the utility of CDSS in LMICs, particularly for critical illnesses. This is unfortunate as the low clinician-to-patient ratios and disparities in specialist physician availability commonly encountered in many LMICs makes the use of CDSS in these settings particularly appealing. A 2022 systematic review of CDSS in LMICs found that to date, the majority of these tools have not been evaluated prospectively, and most have focused on maternal care and infectious diseases, while CDSS for sepsis and non-communicable diseases were identified as priority needs in LMICs [13].

As HICs increasingly benefit from the rich insights available from machine learning approaches applied to patient data, research integrating these approaches for patients in LMICs has not been equitably realized. A 2020 study in the United Kingdom found that a ML automated approach to identify children experiencing significant clinical deterioration via data from wireless monitoring devices performed substantially better compared to traditional pediatric early warning scores (77–97% versus 35% detection of significant events)[23]. Among the few studies using ML approaches in LMICS, a 2019 study evaluated a ML-based triage tool for children in a rural district hospital in Rwanda found strong performance for prediction of in-hospital mortality (AUR 0.8), supporting the promise of these approaches for patient populations in LMICs. Additional studies are also ongoing at the time of this writing, including a multi-site prospective observational study across six Asian countries to build a prediction model for identifying severe illness and mortality among febrile children in LMICs [24].

Wearable sensor technology has continued to evolve, are used for physiological monitoring and are typically multi-parametric, with the ability to measure vital signs, ECG, as well as other features.

These devices are well-suited to use in resource-limited settings as they are relatively low cost (with costs expected to decrease as technology advances, demand increases, and newer reusable patches enter the market) and often require only a mobile device (phone or tablet) for operation. Additionally, ML algorithms which can harness the large amounts of data collected from wearable devices, can now be run on standard smartphones, and allow for point-of-care high-quality monitoring in places with previously insurmountable infrastructure constraints.

While the potential of wearable biosensors to improve patient care in LMICs has been well recognized, few studies have evaluated wearables among real patients in these settings, particularly among children [25,26]. However, in a recent survey of clinician perspectives on wearables in two West African countries, respondents saw great potential in use of these technologies to reduce the time spent manually measuring vital signs, at a significantly reduced cost to bedside monitors [26]. As such, durability, cost, hospital setting, and staffing were all viewed as important contributing factors for wearable sensors to be successfully implemented in LMICs. A 2019 study among children in a Rwandan emergency department (by members of this study team) found that wearable measurements were accurate and feasible for HR and RR, while a 2022 study in Kenya found that a wearable sensor was clinically feasible for HR and temperature measurements among neonates [27,28]. Only one study to date, conducted in 2022 among adult sepsis patients in Vietnam, has developed ML models that were trained on data from wearable sensors and bedside monitors and found strong performance (AUC 0.83) for mortality prediction [29].

While this study was not designed to assess potential implementation barriers to future scale-up, the research team, who had no prior experience with wearable devices or mobile health technology, was able to successfully collect high-quality, continuous physiologic data on all patients in a real-world hospital setting in Bangladesh. The remote transmission to a local smartphone as well as to a Cloud server also allows monitoring from anywhere in the world, enabling consultants to weigh in on patient management from another ward, or even from outside the hospital or country. Based on these preliminary findings, this system may be a viable option for clinical use as a practical, wireless, and portable telemetry monitor for pediatric critical care, although further research is needed to ensure clinical utility in the settings in which it is planned to be used.

### Limitations and future directions

This study was conducted at a single hospital in urban Bangladesh, with personnel with specialty training in pediatrics and critical care, which limits generalizability to other settings. It is important to note that clinical use of these models is not recommended until the models can be externally validated; this research team is currently planning an external validation study amongst a new cohort of children with sepsis in Bangladesh, with future plans to validate the models in other countries and more diverse sites in Bangladesh. Research is also needed to assess implementation barriers and facilitators to scale-up of similar wearable and mobile technology in Bangladesh as well as other LMICs. This technology may also be highly valuable in resource-limited settings within HICs, particularly in rural and hard-to-reach settings. This team is currently developing an implementation science research project to evaluate technology acceptance of this tool among HCWs in district hospitals and rural sites in Bangladesh.

Given this study was conducted in 2022, the revised 2024 sepsis definitions based on Phoenix criteria were not used, as the SIRS-based sepsis criteria were still considered standard practice at that time. SIRS criteria also remain widely used in both HIC and LMICs during early sepsis care due to a lack of laboratory and other clinical values needed for prognostic scores. Model development using the Phoenix criteria as an outcome are planned by this study team. While the majority of the variables in Model B and C were wearable-derived, Model B used MAP and SpO2:FiO2 which are components of the pSOFA score; this may have caused optimistic model performance estimates due to incorporation bias. However Model C uses no variables part of the pSOFA score and may have the highest utility in resource-limited settings given its lack of requirement for any additional clinical inputs aside from the wearable device.

While clinical documentation at the study site is performed on a regular basis by experienced clinicians, it is important to understand that the most pragmatic comparator for diagnosis of advanced sepsis was the EHR clinical documentation, and not the real-time diagnosis by actual clinicians. Delays in clinical documentation are common in clinical settings everywhere, particularly in busy hospital wards, so it is probable that the documentation of advanced sepsis was likely later than actual clinician recognition of advanced sepsis. While research staff were asked to have clinical staff communicate to them the estimates of the actual times they clinically diagnosed advanced sepsis, it is probable that some delays in documentation still occurred. Due to the relatively small cohort sample size, multiple observations and data collection points were measured on each child in order to increase the datapoints available for training, contributing to correlation between observations and therefore may have introduced additional bias. Lastly, there were more male than female children in this study, which is in line with prior research from this study site that has found higher rates of diarrhea in male children [30].

Research is currently underway to externally validate the models developed, as well as conduct pre-implementation research to understand how to best develop this technology into a smartphone-based clinical alert system which can provide clinicians with assessments of sepsis severity, supporting high-quality critical care monitoring capacity for pediatric sepsis in resource-limited settings. By allowing clinicians to detect subtle early physiologic changes indicating clinical deterioration, in the future this tool may allow clinicians to intervene earlier, better direct care, and more rationally allocate scarce resources, all without the need for laboratory diagnostics or critical care infrastructure.

## Supporting information

S1 Table Abnormal vital sign thresholds according to modified pediatric early warning score. (DOCX)
